# Transcription Factors as the “Blitzkrieg” of Plant Defense: A Pragmatic View of Nitric Oxide’s Role in Gene Regulation

**DOI:** 10.3390/ijms22020522

**Published:** 2021-01-07

**Authors:** Noreen Falak, Qari Muhammad Imran, Adil Hussain, Byung-Wook Yun

**Affiliations:** 1 Laboratory of Plant Functional Genomics, School of Applied Biosciences, Kyungpook National University, Daegu 702-701, Korea; noorfalak.pk@gmail.com (N.F.); mimranbot@gmail.com (Q.M.I.); 2 Department of Medical Biochemistry and Biophysics, Umea University, 90187 Umea, Sweden; 3 Department of Agriculture, Abdul Wali Khan University, Mardan, Khyber Pakhtunkhwa 23200, Pakistan; adilhussain@awkum.edu.pk

**Keywords:** nitric oxide, transcription factors, gene regulation, plant defense, guard hypothesis

## Abstract

Plants are in continuous conflict with the environmental constraints and their sessile nature demands a fine-tuned, well-designed defense mechanism that can cope with a multitude of biotic and abiotic assaults. Therefore, plants have developed innate immunity, *R*-gene-mediated resistance, and systemic acquired resistance to ensure their survival. Transcription factors (TFs) are among the most important genetic components for the regulation of gene expression and several other biological processes. They bind to specific sequences in the DNA called transcription factor binding sites (TFBSs) that are present in the regulatory regions of genes. Depending on the environmental conditions, TFs can either enhance or suppress transcriptional processes. In the last couple of decades, nitric oxide (NO) emerged as a crucial molecule for signaling and regulating biological processes. Here, we have overviewed the plant defense system, the role of TFs in mediating the defense response, and that how NO can manipulate transcriptional changes including direct post-translational modifications of TFs. We also propose that NO might regulate gene expression by regulating the recruitment of RNA polymerase during transcription.

## 1. Challenges to Plants from Pathogens

Plants are the primary producers of the ecosystem and, due to their sedentary nature, are exposed to various environmental adversities such as cold, heat, flood, salinity, and drought. However, the greatest threat that plants face is the attack from phytopathogens, herbivory, and human activity. Diseases caused by phytopathogens can drastically reduce crop productivity and yield, which affect not only the production of food but also human development [1]. Although technological advancements and scientific contributions have dramatically reduced the losses in yield and productivity, plant diseases still contribute about 20–30% loss in actual production every year [2,3]. This reflects a lack of knowledge of disease management, the mechanisms behind epidemic development, and of the causal agents.

Plant disease results from complex interactions between various biotic and abiotic stressors, including pathogens, hosts, and the environment [4]. Plant pathogens employ multiple approaches to ensure their success. Pathogenic bacteria access the host plant via stomata, hydathodes, or wounds and proliferate in the intercellular space (called the apoplast). Similarly, nematodes access the host plant by inserting a stylet directly into the host plant cell, while pathogenic and symbiotic fungi and oomycetes penetrate plant cells by inserting haustoria [5]. All these diverse pathogen types release effector molecules for their survival. Therefore, a proper understanding of the causal agents and their mechanisms of action is of paramount importance.

## 2. Plant’s Strategy “Guard” and “Decoy” Models

Plants have a well-defined, fine-tuned defense mechanism that responds to environmental threats and pathogen attack as demanded by their sessile nature. Recently, defense strategies in plants that are induced by pathogens were reviewed in detail [6]. Here we will briefly mention how a plant responds when a pathogen tries to invade it. Unlike mammals, plants lack portable defenders or adaptive immune system and rely solely on the innate immunity of each cell and systemic signals emerging from the site of infection [7,8]. There are two main branches of plant defense: the first uses transmembrane pattern recognition receptors (PRRs) that perceive signals from different pathogens and respond to microbial- or pathogen-associated molecular patterns (MAMPS/PAMPs) like flagellin [9]; the second branch functions inside the cell, using plant resistance (*R*) genes [7]. The effector molecules of pathogens are recognized by NB-LRR proteins encoded by plant *R* genes, leading to *R-*avr interactions that induce similar defense responses. This can be better explained by the zigzag model of plant defense presented by Jones and Dangl [5]. According to their model, in the first phase of plant defense response, PAMPs are recognized by plant PRRs, leading to the activation of a defense process called PAMP-triggered immunity (PTI) that can restrict further pathogen growth. In the second phase, the pathogens that succeeded in releasing their effector molecules compromise the PTI, leading to a condition called effector-triggered susceptibility. Recognition of effector molecules by the host cells causes effector-triggered immunity (ETI), resulting in disease resistance and restricted pathogen growth. This puts pressure on the pathogens to acquire additional effector molecules and diversify these effectors to suppress ETI. The last phase of the defense response is critical: if the pathogen is successful in adapting to the host *R* genes the plants will be unable to defend themselves against infection.

The plants seem to be smarter here by not involving the *R* genes directly—a strategy that is termed the “guard hypothesis” [7]. This hypothesis suggests that R proteins recognize pathogen effectors indirectly. For example, RPM1-interacting protein 4 (RIN4) is a plasma membrane–associated protein that is guarded by NUCLEOTIDE BINDING SITE LUCIN RICH REPEAT (NBS-LRR) proteins [5]. It is manipulated by three different types of bacterial effectors. Two effectors (type III), AvrRpm1 and AvrB, interact with RIN4 and induce its phosphorylation [10]. This modification induce transcription of the RPM1 NBS-LRR protein. A third effector, AvrRPt2, is a cysteine protease that cleaves RIN4 at two different sites [10,11]. This cleavage induces the RPS2 NBS-LRR protein [12]. Both RPS2 and RPM1 require NON-RACE-SPECIFIC DISEASE RESISTANCE1 (NDR1) protein, which interacts with RIN4 for resistance against *Pseudomonas syringae*. Interestingly, functional genomics studies have suggested that RIN4 is not the only target of these three effectors [13]. Reports suggest that an effector contributes to virulence by possible manipulation of several other host targets. However, the contact of any of these targets with the effectors is sufficient to activate plant *R* genes. It seems that RIN4 negatively regulates the two NBS-LRR proteins, RPS2 and RPM1. But in *rpm1rps2* (knockout for RPM1 and RPS2) plants, the pathogen effectors AvrRPt2 and AvrRpm1 manipulate RIN4 to suppress PTI [14]. Therefore, the plants use R proteins to guard against pathogens by interacting with the released effectors.

Plant scientists, specifically those who have an interest in evolution, have proposed another model to explain “guard” and “effector” interactions. They called it the “decoy model” [15]. van der Hoorn and Kamoun [15] gave a realistic explanation of two opposing yet unstable naturally selective forces on the guarded effector target. They explained that plant *R* genes are polymorphic, suggesting the presence/absence of functional *R* genes in different individuals of a plant population. In the absence of a functional *R* gene, the binding affinity of the guardee to the effector is compelled to decrease by natural selection, thereby avoiding detection and alteration by the effector. However, natural selection is expected to favor guardees-effector interactions for better pathogen perception in the presence of a functional *R* gene. They suggested that these two opposing pressures on the same effector are an evolutionarily unstable situation that could be eased upon by the evolution of a host protein, which they termed the “decoy,” that could potentially perceive the effector by the R protein without functioning in development or resistance to disease [15].

## 3. Transcription Factors (TFs): Modulators of Gene Expression

TFs are regulatory proteins that are responsible for the mechanistic control of gene transcription. Technically, they act as the on/off switch of gene expression and are responsible for the activation and suppression of genes, thereby regulating their function. They are transcribed in the nucleus, translated in the cytoplasm, and returned to the nucleus to search for their targets in the genomic DNA; therefore, they are also called diffusible regulatory molecules [16]. Their re-entry into the nucleus is mediated by nuclear localization sites found in the protein sequences of all TFs [16]. The TFs bind to specific DNA sequences, called *cis*-regulatory elements or TF binding sites (TFBSs), in the promoter region of a gene and have defined DNA-binding domains. TBFs may also be located in the intron region and play regulatory roles. For example, in *Arabidopsis thaliana*, sequences for *cis*-regulatory elements of the floral homeotic gene *AGAMOUS* (*AG*) are located in the second intron [17]. The second intron contains TFBs for two direct transcriptional activators of *AG*, i.e., LEAFY (LFY) and WUSCHEL (WUS), and other putative regulatory elements. TFBs are usually highly conserved and are crucial for DNA binding and used to classify TFs into various groups or families [18], such as MADS, WRKY, or APETALA2/ethylene-responsive factors (AP2/ERF). TFs can also be categorized based on their three-dimensional protein structure and composition, such as basic helix-loop-helix (bHLH), helix-turn-helix, and zinc finger proteins. Sequence-specific TFs are considered vital for the regulation of genes involved in prokaryotic and eukaryotic cellular mechanisms [19]. In prokaryotes and eukaryotes, gene regulation by TFs occurs through different mechanisms: in the former, TFs role is driven by a single protein, while, in the latter, it is a combined process that requires multiple proteins to coordinate and drive gene regulation. The binding of a TF to the promoter of a gene is spatiotemporally dependent. Phillips [18] quoted an interesting example of β-globin (a protein responsible for oxygen exchange in red blood cells) to explain this: the β-globin gene is present in every human cell, but no cell type other than red blood cells expresses this gene. Reddy, et al. [20] studied the beta-globin promoters of different cell types using DNA footprinting. They found that TFs that could bind to beta-globin promoters were only expressed in erythroblasts (immature red blood cells).

TFs have two domains: a DNA-binding domain and an effector domain that regulates interactions with other TFs or proteins necessary for transcription. Most DNA-binding domains are highly conserved within the members of the same family of TFs, while the effector domains evolve more rapidly. TFs mediate many functions, including gene induction, gene repression, and response to signal transduction under various environmental conditions. In this study, we will focus on the regulatory role of TFs in plant defense and how NO plays a role in translating its bioactivity to recruit these TFs.

## 4. Regulatory Role of TFs in Plant Defense

The two interconnected branches of plant defense, PTI and ETI, are the major defense strategies that plants use immediately after pathogen perception [6]. These strategies require well-communicated signal transduction and fine-tuned regulation of gene expression [21,22,23]. TFs play a key role in innate plant immunity, primarily by regulating genes involved in PTI, ETI, and hormone and phytoalexin synthesis and pathways. One of the immediate responses to pathogen infection is transcriptional reprogramming. A study using high resolution temporal transcriptomic analyses in *Arabidopsis* demonstrated that approximately one-third of the genome showed differential expression in response to the necrotrophic pathogen *Botrytis cinerea* immediately after infection [24]. Thus, transcriptional reprogramming of the plant cell demands significant changes in gene expression to favor defense over other metabolic processes such as growth and development [23]. Recent studies also suggested that a metabolic shift is required to mediate the trade-off between growth and immunity to ensure proper resource allocation for plant survival [25,26,27]. Many TF families have been reported to play key roles in transcriptional reprogramming. WRKY, bHLH, AP2/ERF, NAM/ATAF/CUC (NAC), and MYB are the major plant TF families [28] regulating various biological processes including plant defense.

### 4.1. WRKY TFs

The WRKY TFs often called “jack-of-various-trades” [29], are one of the largest TF families in plants [30]. The detailed composition and mode of action of WRKYs are well explored [30,31,32,33]. Here, we will focus on their functional roles, particularly in plant defense.

The regulatory role of WRKYs in plant defense has been extensively studied, particularly in the model plant *Arabidopsis thaliana,* and are reported to have both negative and positive roles in the regulation of plant defense [34]. Reports suggested that WRKYs regulate PAMP-signaling downstream of the mitogen-activated protein kinase (MAPK) signaling cascade [35]. The MAPK cascade plays a vital role in various defense responses—particularly, in sensing PAMPs or ETI [36]. For example, WRKY33 in *Arabidopsis* is reported to have a role in resistance to necrotrophic fungal pathogens *B. cinerea* and *Alternaria brassicicola* [37]. Recent reports using functional genomics revealed that WRKY33 is required for MPK3/MPK6-induced camalexin biosynthesis [38]. They also showed that WRKY33- and pathogen-induced camalexin production was compromised in *wrky33* mutants. They further suggested that WRKY33 is a pathogen-inducible TF that acts as a substrate for MPK3/MPK6 to undergo phosphorylation and mutation. WRKY33 also binds to the promoter of phytoalexin deficient 3 (PAD3), which catalyzes the final step in camalexin biosynthesis [28], and to the promoters of 1-aminocyclopropane-1-carboxylic acid synthases 2 and 6 (ACS2 and ACS6) in response to *B. cinerea* [39]. Global expression profiling of wild type and susceptible *wrky33* mutants in response to *B. cinerea* indicated differential transcriptional reprogramming, suggesting that unidentified targets for WRKY33 might be critical for establishing immunity to this necrotrophic pathogen [40]. Similarly, the closest homolog of WRKY33 in *Nicotiana benthamiana* WRKY8 (NbWRKY8) is also phosphorylated by MAPKs, resulting in the induction of defense-related genes. Furthermore, silencing causes increased susceptibility to the oomycete *Phytophthora infestans* and the ascomycete fungus *Colletotrichum orbiculare* [41].

WRKY TFs are also reportedly involved in ETI and interact with plant R proteins. For example, in barley, mildew resistance locus A10 (MLA10) NB-LRR protein, which confers resistance to powdery mildew, interacts with *Hordeum vulgare* WRKY1 (HvWRKY1) and HvWRKY2 in the presence of the AVR_A10_ effector [42]. Both HvWRKY1 and HvWRKY2 repress basal defenses against the virulent fungus *Blumeria graminis* that causes powdery mildew. Following infection by *B. graminis* (expressing AVR_A10_), MLA10 interacts with HvWRKY1 and HvWRKY2 to activate the defense. Another study reported that rice panicle blast 1 (Pb1), another NB-LRR protein, interacts with *Oryza sativa* WRKY45 (OsWRKY45), mediating the resistance to rice blast caused by the fungus *Magnaporthe oryzae* [43]. Similarly, in *Arabidopsis*, WRKY52, also called resistance to *Ralstonia solanacearum* 1 (RRS1), is a TIR-NB-LRR protein with a WRKY domain that shows resistance to the bacterial pathogen *Ralsotonia solanacearum* [44]. Using map-based cloning and natural variation analysis, Narusaka, et al. [45] reported that RRS1 interacts with RPS4 for dual resistance toward fungal and bacterial phytopathogens. Similarly, *Arabidopsis* WRKY8 (AtWRKY8) negatively regulates basal defenses to *Pseudomonas syringae* pathovar tomato (*Pst*) while positively regulating defense responses to *B. cinerea* [46].

### 4.2. bHLH TFs

The bHLH TF family reported in animals and plants in 1989 [47,48] and yeast in 1990 [49] comprised of a group of TFs characterized by the so-called “basic helix-loop-helix (bHLH)” domain. The proteins with this domain are known for a broad spectrum of functions that are reviewed in detail by Heim et al. [50]. Here we will briefly discuss their role in plant defense. The bHLH domain comprises an N-terminal stretch of hydrophilic basic amino acids followed by an HLH domain predicted to have amphipathic α-helices with an intervening loop in between, to form dimers [51]. In essence, bHLH TFs bind with E-box sequences (CANNTG) in the promoters of their target genes with variation in binding specificity [52,53]. Studies in mammals have shown that the conserved HLH structure is critical for the formation of bHLH protein dimers [54]. The specificity for a particular protein partner is determined by the α-helices. In *Arabidopsis*, the bHLH TF family includes about 160 members (https://www.arabidopsis.org/browse/genefamily/bHLH.jsp). However, only a few of them have been characterized in detail, which has shown that the bHLH might not be directly involved in plant defense, but they have an indirect connection by producing certain metabolites that are required during stress conditions. For example, in *Arabidopsis*, IAA-LEUCINE RESISTANT3 (ILR3 or BHLH105) represses the production of aliphatic glucosinolates and secondary metabolites produced in response to wounding, insects, or other microbial pathogens [55]. Furthermore, they interact with JA signaling pathway, thus regulating phytohormonal balance which is also critical for plant defense [56]. Song et al. [57] identified members of the bHLH TF family (bHLH3, bHLH13, bHLH14, and bHLH17) to be targeted by JASMONATE-ZEM-Domain (JAZs). Using the loss of function mutants for these bHLH TFs, they showed that bHLH mutants showed sensitivity to JA-inhibited root growth and an increase in JA-induced defense against pathogen infection and insect attack. The transgenic plants overexpressing bHLH13 or bHLH17 showed reduced JA-mediated responses [57]. Another bHLH TF, HBI1 negatively regulates genes that are involved in plant immunity and inhibits PAMP-induced growth arrest thus mediating the trade-off between growth and PAMP-triggered immunity [26]. Similarly, another bHLH TF, ILR3 was reported to regulate iron deficiency, glucosinolate biosynthesis, and pathogen response [55,58]. MYC2 another bHLH TF, regulates a subset of plant defense responses in *Nicotiana attenuate* [59]

### 4.3. AP2/ERF TF

The AP2/ERF is another important plant-specific TF family that regulates stress responses in plants, mostly studied for responses to abiotic stresses [60]. Members of this family are characterized by the presence of an AP2 DNA binding domain which comprises 40–70 conserved amino acids [60,61,62]. The AP2/ERF TFs regulate genes involved in various biological processes including growth and development, hormone signaling, stress responses both at transcriptional and post-translational levels [63,64,65,66]. Studies involving gene expression profiling have shown that most AP2/ERF TFs have a low basal expression and can be induced or reduced by external stress stimuli or hormonal imbalance [67,68]. Some of the important AP2/ERFs include DEHYDRATION-RESPONSIVE ELEMENT BINDING proteins (DREBs), members of the RAP2 family, and ABA INSENSITIVE 4 (ABI4), etc. Reports suggested that AP2/ERFs are induced by the *cis*-regulatory elements present in their promotors. These elements include HEAT SHOCK ELEMENT (HSE), ETHYLEN INSENSITVE 3 (EIN3) BINDING SITE (EBS), LOW-TEMPERATURE RESPONSIVE ELEMENT (LRT), and ABA Response Element (ABRE) [69].

Post-translational changes such as phosphorylation also affect the activity and abundance of AP2/ERFs. Other studies have shown that phosphorylation affects AP2/ERF protein stability and transactivity [69]. For example, in *Arabidopsis*, the positive regulator of ABA signaling pathway SNF1-related protein kinases (SnRKs) interacts and phosphorylates RAV1 to constrain its transcription repression role [70]. Similarly, ERF6 and EFR104 are phosphorylated by mitogen-activated protein kinases (MAPKs) for positive regulation of pathogen responses [71,72]. AP2/ERFs are also characterized in plant defense. Mase, et al. [73] showed in *Arabidopsis thaliana*, by using a structural analog of AAL, a phytotoxin produced by *Alternaria alternata* [74], that the MODULATOR of ALL CELL DEATH 1 (MACD1), and AP2/ERF TF, was involved in ALL-induced cell death and acted downstream of ethylene.

ERF is one of the large subfamilies of AP2/ERFs. In *Arabidopsis thaliana*, there are about 145 members of the AP2/ERF family [67]. Among them, about 65 members are identified as ERFs. Members of the ERF sub-family are characterized for their role in plant defense. In tomato, the Pti4 and Pti5 (ERFs) are phosphorylated by Pto protein when challenged by the virulent *P. syringea*. The *Pst*-induced phosphorylation increases Pti4 and Pti5 binding to their target sequences in defense-related genes [75]. Similarly, tomato ERFs Pti4, Pti5, and Pti6 when overexpressed in *Arabidopsis,* induced defense response, and contributed to resistance against *P. syringae* [76]. In *Arabidopsis* constitutive expression of ERF1 has been shown to increase resistance against several necrotrophic fungal pathogens. [77]. Besides, the ERF1 is considered a point of integration between JA and ethylene signaling pathways. A detailed review on the role of AP2/ERF TFs has reported that members of ERFs are enriched in genes regulating disease resistance pathways [78] suggesting the significant role of this subfamily in the regulation of plant defense responses.

### 4.4. MYB TF Family

MYB TF family is one of the largest and most functionally diverse families and is conserved among all eukaryotes. They are also diverse in their structure and are classified based on the presence of a conserved MYB domain that contains two or three imperfect repeats (R1, R2, and R3). The structure, classification, and functional diversity of MYB TFs have been well studied [79,80,81,82]. The first plant MYB TF was identified in *Zea mays* [83]. Since then, MYB TFs in several other plant species, including *Arabidopsis* [84], have been reported. Although MYB TFs are often implied to be a major player in flavonoid biosynthesis or abiotic stress [85,86,87,88,89], the first MYB gene identified was the oncogene *v-myb* (initially called *mab* or *amv* after the name of avian myeloblastosis virus but later renamed *v-myb*) from the avian myeloblastosis virus [90,91,92]. Hence, their role in disease resistance cannot be ignored.

Hypersensitive response (HR), a form of programmed cell death (PCD), is one of the most effective defense strategies of the host plant in response to pathogen infection. MYB TFs are reported to positively regulate the HR response. Daniel, et al. [93] showed that, in response to avirulent pathogens such as *Xanthomonas campestris* pv campestris, AtMYB30 showed a rapid and transient expression. Functional genomics study using *Arabidopsis Isd* mutants and their corresponding suppressor *phx* mutants, Daniel, et al. [94] reported that MYB30 expression is likely more responsible for the initiation of the HR than for its propagation. Furthermore, overexpression of MYB30 in transgenic plants accelerated the HR following avirulent bacterial pathogen infection and caused HR-like responses to virulent bacterial pathogens [95]. Raffaele, et al. [96] reported that AtMYB30 regulated HR using long-chain fatty acids and their derivatives. Using microarray analyses of *Arabidopsis* plants overexpressing MYB30 (*MYB30 ox*) or antisense (*MYB30 as*), they reported that MYB30 putatively targeted genes encoding the four enzymes forming the acyl-coA elongase complex that synthesizes very-long-chain fatty acids [96]. Reports have suggested that AtMYB60 and AtMYB96 act through an ABA-signaling cascade, while AtMYB96-mediated ABA signals induce pathogen resistance responses by inducing salicylic acid (SA) biosynthesis in *Arabidopsis* [97]. Similarly, AtMYB102/AtM4 and AtMYB41 regulate plant resistance toward the herbivorous insect, *Pieris rape* [98]. Some MYB TFs regulate both biotic and abiotic stress; for example, *AtMYB108,* also called the *Botrytis* Susceptible 1 (BOS1), which is an R2R3 type MYB [99]. MYB TFs are also reported to contribute to systemic acquired resistance (SAR), a type of plant defense in which the signals broadcast from the site of infection to systemic tissues to warn them of the pathogen attack. Segarra, et al. [100] reported that defense pathways triggered by beneficial *Pseudomonas* and *Trichoderma s*pp. strains are very similar and that MYB72 functions as an early point of convergence in the signaling pathways induced by these two different species of microorganisms.

However, it seems that MYB TFs are less studied for their role in plant defense compared to other TF families. Microarray- and RNA-seq-mediated studies can be used to identify the candidate MYB TFs that induce defense responses.

### 4.5. NAC TF Family

The NAC TF family is a key plant-specific TF family. NAC TFs are characterized by the NAC domain, which has a 150 amino acid conserved domain at the N-terminus, and a diversified C-terminal transcription regulatory region (TR) [101]. Some NAC TFs also have a transmembrane domain within the TR domain. The NAC domain has been sub-divided into five sub-groups from A to E [102]. Genome-wide identification of TFs suggested the presence of NAC TFs in many plant species [103,104,105,106,107].

Like other TFs, NACs also have the DNA-binding ability and can regulate abiotic and biotic stresses, growth, and development. For example, cold-induced NTL6, a plasma membrane-bound NAC TF that is involved in the proteolytic activation of the plasma membrane in *Arabidopsis* [108], is reported to bind directly to the promoter of PR genes to induce resistance against pathogens. Similarly, another NAC TF, ATAF1, that is induced by drought, high salinity, ABA, methyl jasmonate, and wounding has multiple functions in *Arabidopsis* [109]. Reports suggested that overexpression of ATAF1 not only enhances drought tolerance but also increases susceptibility to *B. cinerea,* suggesting possible crosstalk between the stressors. Similarly, in rice, JASMONIC ACID 2 (JA2) and JA2-like (JA2L), the two homologous NAC TFs are reported to mediate pathogen-induced stomatal regulation [110], which are considered to be SA- and ABA-dependent processes. These studies suggest that NAC TFs act as interlinking entities in signaling cascades in response to multiple stressors.

Some NAC TFs also act as negative regulators in plant defense responses and are targeted by pathogens to increase susceptibility. As an example, HopD1, a type III effector from *P. syringae*, interacts with NTL9, a membrane-tethered protein at the endoplasmic reticulum, to suppress ETI responses [111]. Similarly, in a study involving potato (*Solanum tuberosum*), Block, Toruno, Elowsky, Zhang, Steinbrenner, Beynon and Alfano [111] showed that two ER-associated *Solanum tuberosum* NTPs, StNTP1, and StNTP2, interact with an RxLR effector from *P. infestans* to prevent the movement of TFs from the ER to the nucleus and, in doing so, suppress defense responses. Similarly, a type III effector from *P. syringae,* HopD1, interacts with membrane-tethered NTL9 to suppress ETI responses [111]. A similar situation was also found in viral pathogenicity, where the TMV replicase protein interreacted with ATAF2, which is an NAC TF, to suppress the basal host defense [112].

Other reports suggested positive regulation of plant defense by NAC TFs. Studies involving RNAi, knockout (KO), and overexpression of genes suggested the role of NAC TFs in various plant-pathogen interactions. NAC TFs are reported to positively regulate plant defense responses by activating PR-related genes and inducing HR at the infection site [108,113,114,115]. The ATFAF1 and its ortholog in barley, HvNAC6, is reported to positively regulate penetration resistance toward the biotrophic fungus *Blumeria graminis* [114,115]. Thus, NAC TFs appear to be key elements in connecting signal transduction cues from different stressors and can be used to relay between various stresses in plants.

## 5. Evolution of Signaling Molecules

The evolution of plants from unicellular organisms to complex multicellular structures demanded the evolution of aerobic metabolisms such as respiration and photosynthesis. These metabolic processes resulted in the generation of reactive oxygen species (ROS) commonly known for causing oxidative damage to proteins, DNA, and other macromolecules such as lipids [116]. However, recent studies have indicated that ROS can act as signaling molecules for regulating various physiological responses such as growth and development [117], abiotic stress responses [118], plant responses to pathogens [119], and stomatal regulation [120]. ROS are produced by the activation or reduction of oxygen and includes the singlet oxygen (^1^O_2_), hydrogen peroxide (H_2_O_2_), superoxide radical (O_2_^−^), and hydroxyl radical (HO·) [121]. Other plant-like organisms constantly produce ROS in organelles like chloroplast, mitochondria, and peroxisomes, as they are the sites for aerobic metabolism. The generation of different ROS in plants is triggered by various environmental and biotic stressors such as drought, salinity, extreme temperature, nutrient deficiency, and pathogen attack [121].

Production of reactive oxygen intermediates (ROIs), primarily O_2_^−^ and H_2_O_2_, collectively termed as the oxidative burst at the site of attempted invasion, is one of the most rapid responses of the plant following pathogen perception [122]. In plants, the oxidative burst was first reported by Doke [123], who noticed the generation of O_2_^−^ following inoculation with an avirulent strain of the fungal pathogen, *Phytophthora infestans.* However, a virulent strain of the same pathogen was unable to induce O_2_^−^ generation. Since then, O_2_^−^ production has been identified in various plant-pathogen interactions involving avirulent bacteria, fungi, and viruses [124]. The most important aspect of these redox molecules is the high reactivity caused by their short half-life. For example, the half-life of O_2_^−^ is less than a second and is quickly dismutated either enzymatically by superoxide dismutase [125] to H_2_O_2_ (a relatively stable molecule) or non-enzymatically [124]. Similarly, protonation of O_2_^−^ can result in the production of hydroperoxyl radicals (HO_2_^−^) that can convert fatty acids to toxic lipid peroxides, resulting in membrane injury. Furthermore, H_2_O_2_ can undergo Fenton reactions in the presence of divalent metal ions such as Fe^2+^, thereby producing the hydroxyl radical (OH^•^), which is the most reactive ROI that can induce lipid peroxidation and damage to nucleic acids and proteins [124].

Plants have a sophisticated antioxidant system that involves antioxidants and antioxidant enzymes along with other small molecules to detoxify these ROS or expel them from the cell. Thus, continuous ROS generation and scavenging events are in operation in plants. ROS scavenging is carried out by induction of non-enzymatic antioxidants such as glutathione (GSH), ascorbate, flavonoids, and alkaloids—primarily by ascorbate and GSH [116]. Reverse genetics studies have shown that mutants with perturbed levels of ascorbic acid or GSH are hypersensitive to stress conditions [124]. Thus, a homeostatic status is important for normal metabolism in the plant. An imbalance will lead to oxidative damage.

## 6. The Era of Nitric Oxide (NO)

Initially reported in the 1980s as an endothelium-relaxing factor (EDRF) in the animal system, NO, quickly gained the attention of scientists due to its tremendous signaling and regulatory roles. The identification of NO as a potent endogenous vasodilator by Schmidt and Walter [126] was a point of excitement and interest for biologists. Subsequent investigations revealed that NO is a multifunctional effector that regulates various physiological processes in mammals, including the relaxation of smooth muscles, neural communication, immune regulation, and inhibition of platelet aggregation [126]. Further insights into the functions of NO came after NO synthase (NOS), the enzyme responsible for NO production was identified [127]. Moreover, studies on its chemical properties and chemistry have contributed to understanding the mechanism of NO signaling.

The use of NO is not restricted to animals: in the last couple of decades, extensive research has established the regulatory and signaling role of NO in plants as well. Initially identified in potato tuber tissue to induce phytoalexin accumulation, NO has been known as the main player orchestrating various cellular processes, including regulation of stomatal closure [128]; inhibition of the activity of certain enzymes [129]; reduction of seed dormancy [130]; repression of floral transition [131]; activation of MAPK signaling cascades [132]; stimulation of seed germination [133]; plant growth and pollen tube re-orientation [134]; modulation of the cell cycle [135]); photorespiration and photosynthesis [136]; regulation of plant responses to drought, salinity, and heavy metal stress (reviewed by [137]); and regulation of phytohormonal signaling in plants. For example, NO regulates gene expression involved in the JA signaling pathway [138]. Similarly, ethylene and auxin interact with NO to regulate root growth and development [139,140]. In addition, NO’s role in the SA pathway has been reviewed in detail [141]. The most important regulatory role of NO is during plant defense [142], which we will discuss in detail later. However, the list of NO functions is ever-growing with the understanding of its chemistry and signaling behavior.

## 7. NO Biochemical Properties, Synthesis, and Signaling

NO, one of the smallest diatomic molecules is a gaseous free radical with a comparatively short half-life. Combined with its neutral charge, NO promotes rapid membrane diffusion and has several features that make it perfectly suited for cellular signaling [143,144]. NO has an unpaired electron that supports its high reactivity with oxygen (O_2_), transition metals, thiols, and superoxide (O_2_^−^). NO reacts with oxygen to produce various nitrogen oxide molecules with different profiles [145]). The removal of the unpaired electron in NO produces the nitrosonium cation (NO^+^), while the addition of an electron forms the nitroxyl anion (NO^–^). These different forms of NO have different chemical reactivities [146]). NO in the form of peroxynitrite (ONOO^−^) a particularly destructive molecule within biological systems, reacts with ROIs in the presence of O_2_ [147].

The production of NO in animal systems is well understood. The major route for NO production in animal systems is the conversion of L-arginine to citrulline in the presence of NADPH and O_2_ by three isoforms of the nitric oxide synthase (NOS) enzyme (reviewed by Alderton et al. [148]. It has been known for a long time that plants release NO [149,150]. Reports suggested that NO release correlates with the tissue nitrite level; therefore, it was thought that NO is generated from the reaction between nitrites and plant metabolites [150]. Subsequently, researchers have shown that the release of NO is attributed to in vivo nitrate reductase (NR) activity [151]. Several experiments exploring the idea of NO generation concluded that NR reduces nitrite to NO [152,153,154]. Further research on the chemistry of NR has shown that, in maize, the *K*_m_ for nitrite is 100 µM and nitrate is a competitive inhibitor with a *K*_i_ of 50 µM [154], suggesting that, under normal conditions where nitrate levels are high and nitrite levels are low, NO production from NR would be low. However, under anaerobic conditions when the nitrite levels are increased, NO production can be increased 100-fold [154]. As most of the focus related to NO production at that time was on NR, it was considered the only enzyme involved in NO production and signaling [152,155,156]. However, there were also arguments against it [157,158].

L-Arginine analogs like *N*-nitro-L-arginine methyl ester (L-NAME) are inhibitors used to block animal NOS. Similar approaches have shown that deploying L-NAME in plants significantly reduces the production of NO, suggesting the presence of a similar enzyme in plant systems [159]. This hints towards the presence of an arginine-dependent NO production mechanism in plants analogous to the one present in animals [160,161,162,163,164]. This school of thought was supported by immunological experiments that suggested that anti-mammalian NOS antibodies cross-reacted with plant proteins; however, proteomic analyses revealed that these proteins are not related to NOS but are heat shock proteins and glycolytic enzymes [163,165]. Although standard animal-like NOS enzymes have been found in lower pants such as the alga *Ostreocuccus tauri* [166]), despite the completion of several plant genomes and decades of research, a canonical plant NOS could not be identified in higher plants. A gene in *Arabidopsis* (*At3g47450*) was reported to encode AtNOS1 and had 16% similarity with a snail NOS [167]. A functional genomics study of this gene using a T-DNA insertion mutant showed that this protein has a key role in NO synthesis in *Arabidopsis* [167]. However, subsequent investigations showed that AtNOS1 was not directly involved in NO synthesis; rather, it was shown to be a GTPase and was renamed as AtNOA1 for “NO-associated 1” [168]. The mystery remains and is a point of interest for many plant biologists.

## 8. The Role of NO in Plant Defense

It is now a widely accepted fact that the most effective weapon of the plant against pathogen attack is the intentional execution of infected cells, termed the HR [5,169,170]. This phenomenon is thought to restrict biotrophic pathogens’ invasion into other parts of the host. However, despite the potential importance of this defense tactic, the underlying mechanism is largely unknown. Emerging evidence suggests that one of the immediate responses of plants after pathogen perception is the generation of NO bursts [142,171]. In plants, this phenomenon was first recorded in soybean during resistance (*R*) gene-mediated defense against *Pseudomonas syringe* pv. *glycinea* expressing the *avrA* avirulence gene in a soybean suspension culture [142]. Kinetic studies suggested that, during plant-pathogen interaction, maximal NO accumulation occurred 4 to 6 h after *R* gene recognition [142,171]. Furthermore, the use of animal NOS inhibitors finally revoked pathogen-triggered NO production [142,171]. Several reports suggested that, in plants, NO plays a major role in the development of hypersensitive cell death and plant disease resistance. It is suggested that HR-mediated cell death is dependent upon the balanced production of NO and ROS [172].

To elaborate on the HR, plant pathologists have compared it to the mechanistic commonalities with the well-explored process of PCD, termed apoptosis. It would dilute the subject matter to extensively discuss apoptosis here; however, several good reviews have discussed it in detail [173,174,175]. Briefly, the key to apoptosis is the activation of cysteine-dependent aspartate-specific proteases (or caspases) that have a wide range of cellular targets [176]. Mur, et al. [177] have explained the relationship between NO and HR. NO signaling is sometimes mediated by ROS; for example, NO in the presence of oxidative damage may associate with the formation of potent peroxynitrite (ONOO^−^). Thus, NO can influence apoptosis in several ways, including through ONOO^−^. Reports suggested that high ONOO^−^ levels can cause severe damage to nucleic acids [178] and that NO has the potential to bind reversibly with the heme group in cytochrome oxidase to restrict electron transport [179], resulting in increased O_2_^−^ and ONOO^−^ production, which culminates in cellular damage [180].

In plants, *R/*avr interactions leading to the HR share several commonalities with animal apoptosis [181]. HR-mediated cell death and the associated calcium influxes result in permeability transition pores and the release of cytochrome *c* in the mitochondria [182]). Like in animals, balanced production of NO and ROIs is important for the induction of cell death in plants [172]. However, unlike in animals where ONOO^−^ has a key role in apoptosis, reports suggested that plants are relatively resistant to this molecule [172] and that H_2_O_2_ plays a key role in developing cell death during HR. In plants, NO interacts with H_2_O_2_ rather than O_2_^−^ due to the acceleration of O_2_^−^ dismutation to H_2_O_2_ by superoxide dismutase (SOD) [172]. This was confirmed by Zago et al. [183] who performed experiments using transgenic tobacco lines with reduced catalase activity. They showed that, after infiltration of NO under moderate light intensities, these transgenics accumulated H_2_O_2,_ and showed significantly increased cell death compared to wild type lines. In another study, the transgenic lines containing a bacterial NO dioxygenase transgene that converts NO to NO_3_ accumulated significantly less H_2_O_2_, suggesting that NO is required for H_2_O_2_ accumulation during HR [184]. However, the molecular mechanisms underlying the interaction between NO and H_2_O_2_ remain unknown.

Emerging evidence suggests that NO not only helps in developing HR but also in the establishment of disease resistance. The first direct link in this context was provided by Delledonne et al. [142], who reported that infiltration of the NOS inhibitors L-NNA and PBITU increased growth of the avirulent bacterial pathogens *P. syringae* pv. *tomato* (*Pst*) DC3000 expressing the *avrRPm1* avirulent effector, suggesting the role of NO in *R*-gene-mediated disease resistance against pathogenic bacteria.

The controlled use of NO donors, in cell suspension cultures of tobacco plants, induces the expression of defense-related genes, encoding pathogenesis-related protein 1 (PR1), phenylalanine ammonia-lyase marker for phenylpropanoid biosynthesis, and SA mediated signaling. Both genes play a valuable role in the growth and development of plants’ disease resistance [142,171]. *R* proteins in plants, produced on pathogen recognition, trigger the inducible defense response [7]. In the absence of *R* gene recognition, plants depend on their basal resistance responses.

It has been proposed that NO functions in basal disease resistance that is triggered by the recognition of lipopolysaccharides (LPS) [185], which exhibit a pathogen-associated molecular pattern (PAMP) [186]. Loss of AtNOA function diminished NO accumulation in response to LPS, reduced defense-related transcript accumulation, and, most significantly, compromised basal disease resistance against *Pst* DC3000 [187]. Collectively, these data argue that NO has an important signaling function in basal disease resistance—at least against bacterial pathogens.

## 9. NO and TFs

Due to its high reactivity and unique chemistry, NO and its derived redox-active species are excellent biological messengers in plants and animals. Despite the importance of NO in various cellular processes, its mode of action remains poorly understood. Scientists have tried to explain it using NO-mediated redox modifications that have the potential to regulate protein function. One such mechanism is the post-translational modification, in which an NO moiety is covalently attached to exposed cysteine thiols, making S-nitrosothiols (SNOs) [188]. Reports suggested that, contrary to other signaling cascades, NO functions by transferring its bioactivity through *S*-nitrosation (previously called *S*-nitrosylation). After cGMP signaling, *S*-nitrosation is the most important feature of NO [189] and plays a key role in cellular processes that modulate enzyme activity, protein localization, and protein-protein interactions [190]. Several proteins regulating key physiological processes have been reported to be *S*-nitrosated by NO, including NPR1 [190], AtSABP3 [191], NADPH oxidase [192], and the auxin receptor TIR1 [193]. Besides, Lindermayr, et al. [194] identified more than 100 other proteins as potential candidates for S-nitrosation in *Arabidopsis*. Similarly, a site-specific proteomic study of *atgsnor1–3* having perturbations in *Arabidopsis S-*nitrosoglutathione reductase (*AtGSNOR*), thus having higher SNO levels [170], showed 926 proteins and 1195 peptides that were *S*-nitrosated [195].

However, the question remains: how does NO regulate expression? The eukaryotic gene expression is modulated by Pol II that require GTFs to bind to the promoter of a gene to enhance or repress its expression. Thus, Pol II recruitment is one of the key events of transcription processes. The TFs may also interact with other proteins and bind to the promoter as a protein complex [189]. However, the DNA-binding affinity of TFs can be altered by redox-mediated post-translational modifications (such as *S*-nitrosation or phosphorylation) that have the potential to bring conformational changes into the protein structure and alter its function. For instance, *S*-nitrosation affects the structure and DNA-binding activity of AtMYB30 in *Arabidopsis* [196]. Similarly, OxyR, a thiol-containing transcriptional activator that, upon oxidation, regulates the expression of genes involved in H_2_O_2_ detoxification, is modulated by *S*-nitrosation [197]. Studies by others have supported our argument by showing that *S*-nitrosation directly modifies several transcription factors, including NF-κB, HIF-1 [198] zinc finger transcription factor SRG1 [199] bZIP TF TGA1 [200]. Studies involving NO-mediated transcriptional changes have shown that a substantial number of genes and TFs are regulated by NO. Changes in cellular redox tone mediated by NO can regulate the expression of important genes and TFs such as HY5, MYB, and Trx [189], suggesting that NO plays a role in the regulation of various cellular processes via mechanistic control of transcriptional machinery. In the model plant *A. thaliana*, transcriptional changes in response to NO have been studied using cDNA-amplified fragment length polymorphisms [201], microarrays, real-time PCR [202,203], and RNA-seq [204,205]. Transcriptome analyses in response to different NO donors have shown differential expression of numerous genes. In an RNA-seq-based transcriptomic approach using *Arabidopsis* roots and leaves, Begara-Morales et al. [204] showed a differential expression of 3263 genes and 35 TFs after 3 h of 1 mM GSNO application. It was interesting that, among the 35 differentially expressed TFs, 25 were from roots and only 10 from leaves. Similarly, in response to 0.1 mM and 1 mM sodium nitroprusside (SNP), Parani, et al. [203] showed differential expression of 422 genes in *A. thaliana* using whole-genome microarray analysis. Recently, using high throughput RNA sequencing, changes in the expression of about 6,436 *Arabidopsis* genes 6 h after infiltration of 1 mM *S*-nitrocysteine (CySNO) were reported [205]. These included about 673 TFs representing a broad range of TF families. Gene ontology and MapMan analyses showed that these genes were enriched in pathways like hormone signaling, protein degradation, and biotic and abiotic stresses [206]. A list of top 20 differentially expressed TFs in response to 1 mM CySNO is given in Table 1 which shows various biologically important TFs such as ABR1 that is expressed in response to ABA or osmotic stress, DREB2C involved in drought stress, AtMYB3, that represses phenylpropanoid biosynthesis gene expression, and AtMYB48 involved in cold stress acclimation (Table 1). The differential expression of this huge number of genes by a single molecule could only be explained by the co-operation of a set of TFs that could bind to a common region in the promoter of the regulated genes [189]. To find this, Palmieri et al. [189] searched for a common TFBS in the promoter region of NO-regulated genes based on microarray analyses using Genomatix, Gene2Promoter, and MatInspector. They found that eight families of TFBSs occur at least 15% more often in the promoter region of NO-responsive genes compared to more than 28,000 *Arabidopsis* genes. Among these, the majority were ocs element-like-sequences and WRKYs. The above-mentioned evidence establishes the mechanistic control of gene transcription by direct pos-translational modification of TFs, thereby affecting their DNA-binding affinity. Table 1. List of top-20 up- and down-regulated transcription factors (TFs) that were differentially regulated in response to 1 mM CySNO. Red and green color represents up- and down-regulated TFs respectively [206].

## 10. Conclusions and Future Recommendations

**Figure 1:**
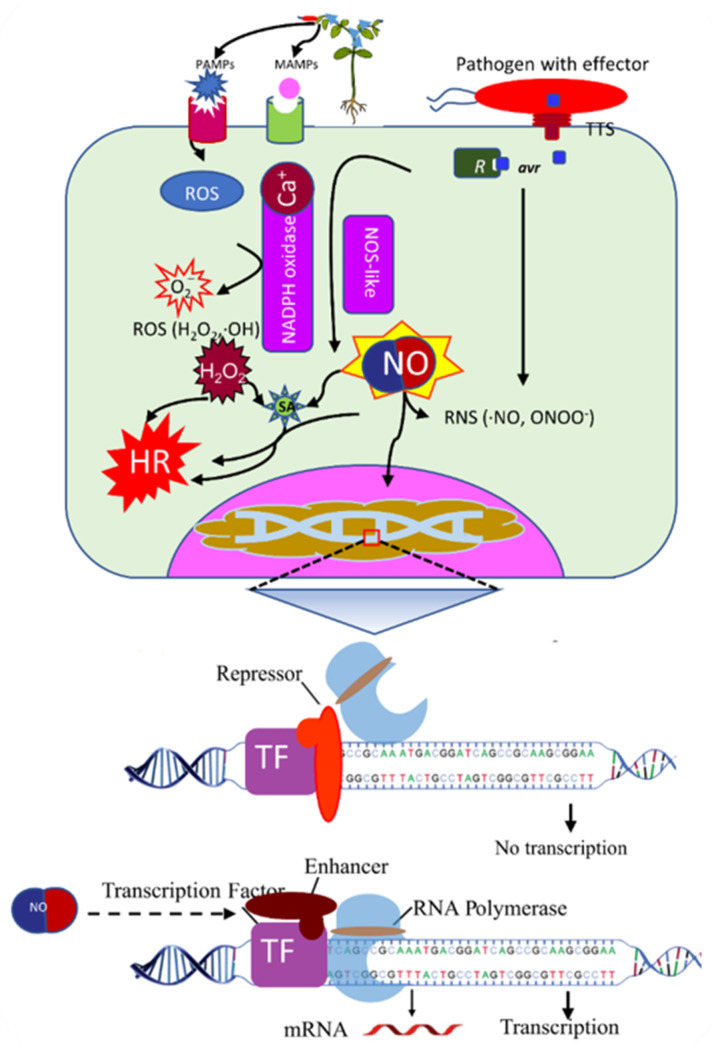
Production of nitric oxide (NO) in response to virulent and avirulent pathogens. Putative model showing the production of NO in response to pathogens, and its role in the recruitment of RNA polymerase under stress conditions. TFs can enhance or repress the expression of genes. PAMP: pathogen-associated molecular patterns, MAMP: Microbe-associated molecular patterns, TTS: type three secretion system, ROS: reactive oxygen species, RNS: reactive nitrogen species, NOS, nitric oxide synthase, NADPH: Nicotinamide adenine dinucleotide phosphate, HR: hypersensitive cell-death response.

Plants, the primary producers of the ecosystem, are under continuous threat from several environmental adversities such as cold, heat, flood, salinity, and drought. Besides, attack from phytopathogens is a serious problem that demands the immediate attention of plant scientists. As part of their adaptation to their sedentary nature, plants have a fine-tuned defense mechanism that responds to environmental constraints and pathogen attack. The NO, initially reported in animal systems as an endothelium-relaxing factor, gained the attention of scientists due to its tremendous signaling and regulatory roles. Research reports in the last couple of decades have unraveled the role of NO in plant defense. After pathogen perception, redox bursts result in the production of NO and ROS inducing a downstream signaling cascade including the induction of HR response and activation of pathogen-induced SA pathway (Figure 1). But how does NO regulate gene expression? One of the mechanisms includes direct *S*-nitrosation of transcription factor proteins by nitric oxide that results in significant changes in the structure of these proteins, thereby affecting their ability to bind at their specific sites in the promoters of target genes.

**Figure 2:**
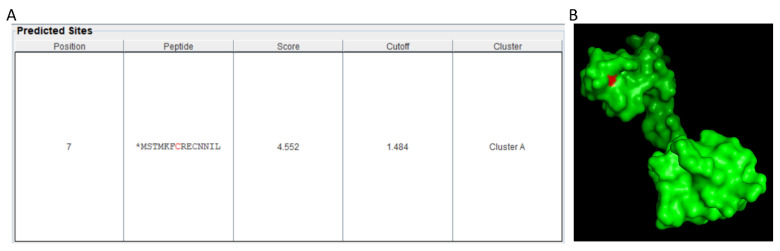
Prediction of possible *S-*nitrosation site in Rpb9 subunit of Pol II. (**A**) The peptide sequence of Rpb9 was analyzed using GPS SNO 1.0 with maximum threshold. Cys07 was predicted as site for S-nitrosation (**B**) The 3D structure of Rpb9 was retrieved from the uniport database and analyzed using PyMOL 2.4. The Cys07 was highlighted as red.

Another possible mechanism may be via modification of the RNA polymerase II by NO. All the eukaryotic genes are transcribed by RNA polymerase II (Pol-II) which is a complex of 12 subunits (Rpb1-Rpb12). However, Pol-II cannot recognize the promoter sequence on its own, rather it requires general TFs (GTFs)—TFIIA, TFIIB, TFIID, TFIIE, TFIIF and TFIIH [207,208] and are conserved across the eukaryotic species including plants [209]. These GTFs along with Pol II assemble in a defined order on the promoter of a target gene to make a pre-initiation complex [210]. Histone integrity is crucial for maintaining these complexes (to maintain its binding to the promoter) for proper functioning. Post-translational modification (PTMs) including acetylation, phosphorylation, and methylation can cause histone modification [211] and hence can hinder transcription. NO can also modulate PTMs, the chief among them is *S*-nitrosation, therefore NO has the potential to mediate RNA polymerase binding and regulate transcription possibly through *S*-nitrosation of one or more of the Pol II subunits or GTFs. To support our argument, we analyzed the protein sequence of *Arabidopsis* Rpb9, a core subunit of Pol II through GPS-SNO 1.0 [212] and found that even using a high threshold, there was a strong prediction for possible *S*-nitrosation of the cysteine residue (Cys 07) (Figure 2A). Further studying the 3D structure of Rpb9, we found that the target Cys07 was also solvent-exposed (Figure 2B) making it a potential target for *S*-nitrosation. However, detailed in vitro and in vivo investigations are required to confirm this hypothesis. A combined approach, using genomics, transcriptomics, proteomics, and metabolomics may be required to unravel the unexplored roles of NO in gene transcription.

## Figures and Tables

**Table 1:** List of top-20 up- and down-regulated transcription factors that showed differential expression in response to CySNO in RNA-seq based transcriptome.

Locus ID	Name	Log2 Fold Change	Annotation
AT1G71520	-	10.8359	Encodes a member of the DREB subfamily A-5 of ERF/AP2 transcription factor family. The protein contains one AP2 domain.
AT2G22760	bHLH	10.1887	Basic helix-loop-helix (bHLH) DNA-binding superfamily protein; FUNCTIONS IN: DNA binding, sequence-specific DNA binding transcription factor activity
AT1G22810	-	9.96553	Encodes a member of the DREB subfamily A-5 of ERF/AP2 transcription factor family. The protein contains one AP2 domain.
AT1G43160	RAP2.6	9.70626	Encodes a member of the ERF (ethylene response factor) subfamily B-4 of ERF/AP2 transcription factor family (RAP2.6). The protein contains one AP2 domain.
AT5G64750	ABR1	9.68623	Expressed in response to ABA, osmotic stress, sugar stress and drought. Mutants are hypersensitive to these stresses. May be involved in regulation of ABA-mediated stress response.
AT3G53600	AT3G53600	8.79631	C2H2-type zinc finger family protein, involved in response to chitin, regulation of transcription
AT4G29930	AT4G29930	8.39629	Basic helix-loop-helix (bHLH) DNA-binding superfamily protein
AT4G28140	-	8.20867	Encodes a member of the DREB subfamily A-6 of ERF/AP2 transcription factor family. The protein contains one AP2 domain.
AT2G40340	DREB2C	7.70854	Encodes a member of the DREB subfamily A-2 of ERF/AP2 transcription factor family. There are eight members in this subfamily including DREB2A AND DREB2B that are involved in response to drought.
AT4G05100	AtMYB74	7.66846	Member of the R2R3 factor gene family.
AT5G01900	WRKY62	7.48636	Member of WRKY Transcription Factor; Group III
AT5G53290	CRF3	7.4745	Encodes a member of the ERF (ethylene response factor) subfamily B-5 of ERF/AP2 transcription factor family.
AT5G01380	AT5G01380	7.40458	Homeodomain-like superfamily protein; CONTAINS InterPro DOMAIN/s: SANT, DNA-binding (InterPro:IPR001005), MYB-like
AT4G37850	-	7.35975	Basic helix-loop-helix (bHLH) DNA-binding superfamily protein; FUNCTIONS IN: DNA binding, sequence-specific DNA binding transcription factor activity
AT1G52890	ANAC019	7.24886	Encodes a NAC transcription factor whose expression is induced by drought, high salt, and abscisic acid. This gene binds to ERD1 promoter in vitro.
AT3G06490	AtMYB108	7.15948	Putative transcription factor MYB108 (MYB108) mRNA,
AT1G22640	ATMYB3	7.12171	MYB-type transcription factor (MYB3) that represses phenylpropanoid biosynthesis gene expression
AT3G50260	CEJ1	7.04121	Encodes a member of the DREB subfamily A-5 of ERF/AP2 transcription factor family. The protein contains one AP2 domain. Involved in defense and freezing stress responses.
AT4G27950	CRF4	7.03719	Encodes a member of the ERF (ethylene response factor) subfamily B-5 of ERF/AP2 transcription factor family. The protein contains one AP2 domain. There are 7 members in this subfamily.
AT4G18170	WRKY28	7.00802	Member of WRKY Transcription Factor; Group II-c. Involved in the activation of salicylic acid biosynthesis genes ICS1 and PBS3.
AT3G52910	AtGRF4	−4.95192	
AT4G32280	IAA29	−4.88034	Encodes a member of the DREB subfamily A-4 of ERF/AP2 transcription factor family. The protein contains one AP2 domain. There are 17 members in this subfamily including TINY.
AT5G03150	JKD	−4.84552	Winged-helix DNA-binding transcription factor family protein; FUNCTIONS IN: DNA binding; INVOLVED IN: nucleosome assembly
AT3G46130	ATMYB48-3	−4.80639	Encodes ICE2 (Inducer of CBF Expression 2), a transcription factor of the bHLH family that participates in the response to deep freezing through the cold acclimation-dependent pathway. Overexpression of ICE2 results in increased tolerance to deep freezing stress after cold acclimation.
AT1G73830	BEE3	−4.50709	Encodes ICE2 (Inducer of CBF Expression 2), a transcription factor of the bHLH family that participates in the response to deep freezing through the cold acclimation-dependent pathway. Overexpression of ICE2 results in increased tolerance to deep freezing stress after cold acclimation.C144:C163
AT3G55734	MIR393B	−4.48027	Similar to a putative transcription factor and transcriptional coactivators. Repressor of GA responses and involved in gibberellic acid mediated signaling. Represses GA-induced vegetative growth and floral initiation. Rapidly degraded in response to GA.
AT1G64625	-	−4.24715	
AT4G30410	AT4G30410	−4.22033	BR enhanced expression 1 (BEE1); FUNCTIONS IN: sequence-specific DNA binding transcription factor activity
AT1G11850	-	−4.18487	Homeodomain-like superfamily protein; FUNCTIONS IN: DNA binding, sequence-specific DNA binding transcription factor activity
AT4G36540	BEE2	−4.1675	B-box type zinc finger protein with CCT domain; FUNCTIONS IN: sequence-specific DNA binding transcription factor activity, zinc ion binding
AT1G49010	-	−4.12299	Dof-type zinc finger DNA-binding family protein; FUNCTIONS IN: DNA binding, sequence-specific DNA binding transcription factor activity
AT4G14540	NF-YB3	−3.96951	Encodes a member of the KANADI family of putative transcription factors. Together with KAN1, this gene appears to be involved in the development of the carpel and the outer integument of the ovule.Along with KAN1 and KAN4 appears to regulate the proper localization of PIN1 in early embryogenesis.
AT3G11090	LBD21	−3.95739	NAC 014 (NAC014); FUNCTIONS IN: sequence-specific DNA binding transcription factor activity; INVOLVED IN: multicellular organismal development
AT1G21150	-	−3.9351	
AT3G61950	-	−3.89039	Encodes a putative MYB domain containing transcription factor involved in anthocyanin metabolism and radical scavenging.
AT2G05160	AT2G05160	−3.82318	Serine/threonine-protein kinase WNK (With No Lysine)-related
AT3G48550	-	−3.79433	Encodes the longer of two splice variants of a transcription factor involved in regulating starch metabolims in response to cold.
AT1G47655	-	−3.79179	B-box type zinc finger protein with CCT domain; FUNCTIONS IN: sequence-specific DNA binding transcription factor activity, zinc ion binding
AT5G15830	bZIP3	−3.74044	
AT5G23920	-	−3.69122	Myb-like transcription factor family protein; CONTAINS InterPro DOMAIN/s: SANT, DNA-binding (InterPro:IPR001005), Homeodomain-like (InterPro:IPR009057)

## Data Availability

No new data were created or analyzed in this study. Data sharing is not applicable to this article
